# A Scintillator Beam Monitor for Real-Time FLASH Radiotherapy

**Published:** 2023-05-24

**Authors:** Daniel S. Levin, Claudio Ferretti, Nicholas Ristow, Monica Tecchio, Peter S. Friedman, Dale W. Litzenberg, Vladimir Bashkirov, Reinhard Schulte

**Affiliations:** 1. Department of Physics, University of Michigan, Ann Arbor, MI, 48109, USA; 2. Integrated Sensors LLC, Palm Beach Gardens, FL 33418, USA; 3. Department of Radiation Oncology, University of Michigan, Ann Arbor, MI, 48109, USA; 4. Division of Biomedical Engineering Sciences, Loma Linda University School of Medicine, Loma Linda, CA 92350, USA

## Abstract

**Background:**

FLASH Radiotherapy (RT) is a potentially new cancer radiotherapy technique where an entire therapeutic dose is delivered in about 0.1 s and at ~1000 times higher dose rate than in conventional RT. For clinical trials to be conducted safely, precise and fast beam monitoring that can generate an out-of-tolerance beam interrupt is required.

**Purpose:**

A FLASH Beam Scintillator Monitor (FBSM) is being developed based in part on two novel proprietary scintillator materials with capabilities that conventional RT detector technologies are unable to simultaneously provide: 1) large area coverage; 2) a low mass profile; 3) a linear response over a broad dynamic range; 4) radiation tolerance; 5) real-time analysis IEC-compliant fast beam-interrupt signal; 6) true two-dimension beam imaging with excellent spatial resolution. This paper describes the design concept and reports results from prototype devices.

**Methods:**

The FBSM uses two types of proprietary low mass (< 1 mm WE), non-hygroscopic, radiation tolerant scintillator materials (designated PM and HM: polymeric and hybrid material, respectively) that are viewed by high frame rate machine-vision cameras. Folded optics using mirrors enable a thin monitor profile of ~10 cm. The scintillator selection is determined by the specific beam type and delivery configuration. An FPGA-based data acquisition system currently under development generates real-time analysis and a beam interrupt signal on a time scale appropriate to the FLASH RT beam modality: 100–1000 Hz for pulsed electrons and 10–20 kHz for quasi-continuous scanning proton pencil beams. Two prototype monitor devices were fabricated and tested in various radiation beams that include heavy ions, low energy protons at nA currents, FLASH level dose per pulse electron beams, and in a hospital radiotherapy clinic with electron beams.

**Results:**

Results presented in this report include image quality, response linearity, radiation hardness, spatial resolution, and real-time data processing. Both scintillator materials were found to be highly radiation damage resistant. PM and HM scintillator exhibited no measurable drop in signal after a cumulative dose of 9 kGy and 20 kGy respectively. HM showed a small −0.02%/kGy signal decrease after a 212 kGy cumulative dose resulting from continuous exposure for 15 minutes at a high FLASH dose rate of 234 Gy/s. These tests established the linear response of the FBSM with respect to beam currents, dose per pulse, and material thickness. Comparison with commercial Gafchromic film indicates that the FBSM produces a high resolution 2D beam image and can reproduce a nearly identical beam profile, including primary beam tails. Double Gaussian fits to the beam profile show that the FBSM and Gafchromic film yield the same fit parameters to within 1.4% of their average values. At 20 kfps or 50 μs/frame, the real-time computation and analysis of beam position, beam shape, and beam dose takes < 1 μs.

**Conclusions:**

The FBSM is designed to provide real-time beam profile monitoring over a large active area without significantly degrading the beam quality. Prototype devices have been staged in particle beams at currents of single particles up to FLASH level dose rates, using both continuous ion beams and pulsed electron beams. Using our novel scintillators, beam profiling has been demonstrated for currents extending from single particles to 10 nA currents. Radiation damage is minimial and even under FLASH conditions would require ≥ 50 kGy of accumulated exposure in a single spot to result in a 1% decrease in signal output. Spatial resolution was comparable to radiochromic films. Real-time data processing, taking < 1 μs, is being implemented in firmware for 10–20 kHz frame rates for continuous proton beams and for pulsed electron beams from 100–1000 Hz.

## 1 INTRODUCTION

FLASH Radiotherapy (RT) is an emerging modality for cancer treatment in which normal tissue toxicity is reduced by the use of ultra-high dose rates exceeding 40 Gy/s. Several animal pre-clinical animal trials used pulsed electron beams [1] [2] [3], while the first human clinical trial (FAST-01) was done with a proton beam [4]. In electron-FLASH, a linac generates a sequence of short 2–4 μs pulses at a repetition rate in the range of 100–1000 Hz [5] [6]. Proton-FLASH consists of passively scattered or scanning pencil beams typically from isochronous cyclotrons, which have a quasi-continuous structure typically consisting of 2 ns duration bunches at a frequency of roughly 70 MHz, or synchrocyclotrons, which produce beams of microsecond pulses with gaps of milliseconds. In any of these FLASH RT delivery modes, the time-averaged dose rate can be at least two or even three orders of magnitude higher than in conventional RT. Notably, the instantaneous dose rate is orders of magnitude higher still, extending up to > 10^9^ Gy/second [7].

Precise and fast real-time beam monitors are required for future patient treatments to be conducted effectively and safely. The quasi-continuous versus pulsed nature of these beams dictates some aspects of the monitor components, as described below. Instrumentation for conventional RT dose measurement includes ionization chambers and passive radiochromic films that provide detailed and precise dosimetry information minutes to hours after exposure. Real-time beam monitoring is typically implemented with strip ionization chambers or inductive beam current transformers, for example, the unit made by Bergoz (Saint Genis Pouilly, France) [8], which is used in the Mobetron (IntraOp, Sunnyvale, CA, USA) medical linac. However, these beam monitoring instruments are not in general optimized for FLASH beam applications as they may saturate or lose efficiency at high rates, can have slow response times, have insufficient spatial resolution, have limited area coverage, or introduce excessive mass in the beam path. Several research programs are exploring FLASH-compatible beam monitors [9] [10] [11] [12] [13] [14]. To the best of our knowledge, no single technology provides the combined attributes of large area coverage, a low mass profile (< 1 mm WE), large dynamic range with a linear response, high radiation tolerance, true two-dimensional imaging with high spatial resolution, and most critically for FLASH operation, the capability to generate a real-time beam-interrupt if the beam current or beam profile deviates from a prescribed irradiation program. This paper presents an NIH/NCI funded FLASH Beam Scintillator Monitor (FBSM) with these capabilities. Experimental results are reported from two prototypes that have been tested at the DOE Facility for Rare Isotope Beams (FRIB), at the Michigan Ion Beam Laboratory (MIBL), at the Notre Dame Radiation Laboratory (NDRL), and at the University of Michigan Hospital (UMH).

## 2 METHODS

### 2.1 Monitor Design

**Figure 1:**
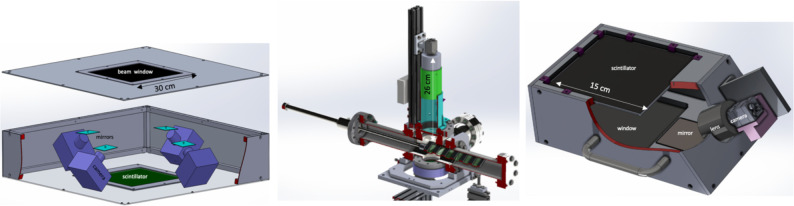
(left) Concept drawing of four-camera FBSM; (center) “pre”-prototype 6-way cross (WC) beam monitor with translatable scintillator cassette; (right) single-camera prototype FLASH beam monitor.

The FBSM employs two types of proprietary application-specific, thin, low mass scintillators (described below) configured into a large 30 cm × 30 cm sensitive area through which the beam passes. The total longitudinal mass profile for the FBSM ranges from 200–700 μm water equivalent (WE). The beam entrance and exit windows are made from opaque/black thin films contributing ~30 μm WE. The low mass ensures that the monitor is beam transmissive with low energy loss and minimal particle scattering. For a 170–250 MeV proton beam, it is practically 100% transmissive, losing only 0.10–0.15 MeV or < 0.1% of its energy and for a 6–16 MeV clinical electron beam, only 0.14–0.16 MeV, or ~1.4% of its energy is lost [15]. The active area of the transmissive scintillator is viewed by four machine-vision CMOS cameras operating in either a triggered shutter or a continuous mode. The triggered image frame mode is intended for electron beams with pulse repetition rates of order ~100–400 Hz at full spatial resolution, while extended operation to 1 kHz pulse rates is associated with reduced spatial resolution. A continuous acquisition mode is planned for scanning proton beams at frame rates extending to 10–20 kHz. In this mode, the spatial resolution is expected to be better than 0.5 mm. The FBSM design utilizes four cameras to cover the full scintillator area. Each camera views a single quadrant of the scintillator via front-aluminized high reflectivity mirrors, with some overlap allowed in the central region, as shown conceptually in the left panel of Figure 1. In addition, an internal, self-monitored UV LED-based calibrator mechanism is included (not shown) to monitor the long-term scintillator stability. Camera image data are routed on high speed (12 Gb/s) copper lines to an FPGA/CPU module. Data pre-processing, including merging of the four camera images, background subtraction, perspective transformations into beam coordinates, dosimetry corrections, and a full beam profile analysis are all performed in firmware in < 1 μs. The real-time analysis output includes beam centroids, RMS widths and the integrated light yield, which is directly correlated to the dose. Absolute dose at the isocenter is calibrated with Gafchromic films using standard clinical protocols [16]. The fast FPGA processor is designed to generate an out-of-tolerance beam-interrupt logic signal with respect to an encoded treatment program.

### 2.2 Scintillator Materials

The two proprietary scintillator materials used in the FLASH monitor program are generically referred to as Polymer Material (PM) and Hybrid Material (HM). Both are non-hygroscopic and exhibit excellent radiation damage resistance and are described below.

#### 2.2.1 PM Scintillator

PM is a semicrystalline polymer developed initially as a physically resilient thin-film sheet primarily for high-volume commercial applications and later discovered to be an intrinsic scintillator. In optical test bench experiments, PM produces a much stronger photodetector signal than other fully transparent plastic scintillators tested, such as polyvinyltoluene (PVT) based BC-400 [17] and, by inference, also polystyrene (PS) based scintillators which were shown to have lower light production efficiency than PVT based scintillators [18] [19]. The stronger signal in PM is largely due to its polycrystalline structure, which gives it a hazy appearance and eliminates internal reflections. More photons escape from the surface towards the camera lens, whereas in clear plastic scintillators, the majority of generated photons internally reflects and preferentially emerges from the edges. PM is an excellent material for the FBSM: it is commercially available in thin sheets (< 200 μm), can cover a 30 cm × 30 cm area, has high tensile strength, is radiation damage resistant, and light emission is uniform over the surface area. We have evaluated PM scintillators with thickness from 200 μm down to 1 μm (< 0.3 mm WE).

#### 2.2.2 HM Scintillator

HM is an inorganic-polymer material that is available in both thin sheets (~0.5–0.8 mm WE) and large area sizes. In test bench experiments using low energy betas, it generates a five-fold larger amplitude signal per unit thickness than unpolished CsI(Tl). It is noted that unpolished CsI(Tl) generates more front-surface emission than polished CsI(Tl). The HM photon production efficiency is estimated to be similar to CsI(Tl) (and much larger than PM or BC-400), which is in the overall range of 48,000–65,000 photons/MeV [20] [21]. Being polycrystalline in nature, it is visually opaque and incapable of total internal reflection, thus resulting in a higher percentage of photons escaping from the front surface and reduced back surface reflections and scattering, especially since it is much thinner than comparative CsI(Tl) plates.

### 2.3 FBSM Prototypes

Two prototype devices have been fabricated and tested. The first one, referred to as the 6-way-cross (6WC) shown in Figure 1 (center), was developed as an ion beam monitor that can be installed in vacuum beam lines [22]. It has an internal cassette of 2 cm × 4 cm scintillators that can be robotically translated in/out of the beam. The camera views the scintillator through an ultrafast f/0.9 lens. The image sensor is a megapixel CMOS with 12 bit ADC digitization. The pixel readout noise is < 3 e^−^ (RMS), corresponding to approximately a single ADC count. The second prototype in Figure 1 (right) is a single machine-vision camera version of the Figure 1 (left) device, referred to as prototype-FBSM. The 15 cm × 15 cm scintillator is viewed through a mirror at an angle. A perspective homography transform matrix converts images into the beam coordinate system. High frame rates required by continuous scanning proton beams are enabled by projecting the entire active scintillator onto a reduced sensor region of interest for high-speed readout. For example, a pixel field of 480H × 320V allows a 15 kHz camera frame rate, corresponding to a 66 μs overall timing budget in which data analysis must be completed in the FPGA. By limiting the sensor field even further, a beam spot spatial resolution < 1 mm can still be achieved at a maximum 20 kHz frame rate. The comparatively slow pulse repetition rates offered by even the fastest electron beams present a less challenging time budget in which to acquire and analyze an image. A spatial resolution of < 100 μm and < 0.5 mm are expected for pulse rates of < 400 Hz and ≤ 1 kHz, respectively.

## 3 RESULTS

FBSM prototypes were deployed in various beam environments. Evaluations of HM and PM scintillators were initially conducted on a test bench using a collimated beam of β^−^ particles from a ^90^Sr source. PM was shown to provide a significantly sharper beam image with larger signals than a commercial standard BC-400 scintillator. Similarly, HM produced a cleaner image, free of reflections and edge smearing effects from photon “blooming”, and stronger amplitude beam signals than a benchmark CsI(Tl) crystal [22]. New results reported here were acquired for PM and HM using the 6WC at FRIB, MIBL, and NDRL, while the prototype FBSM was tested at UMH under conventional RT conditions, and at NDRL under FLASH-level dose rates. Each of these beam environments probed different parameters of the detector operation.

### 3.1 FRIB Beam Test

**Figure 2:**
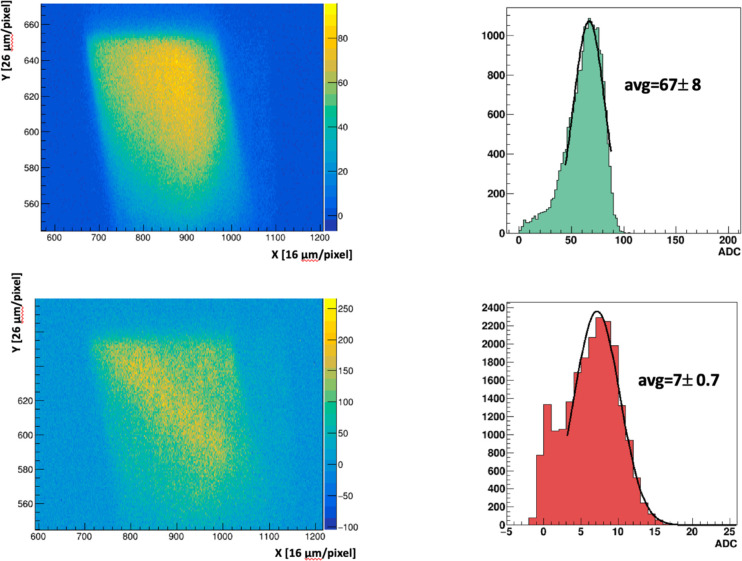
Background subtracted images of the FRIB ^86^Kr^+26^ beam on 6 μm thick PM scintillators at 520K pps (top), 51K pps (bottom). The distance per pixel refers to beam pipe coordinates. The ADC spectra are from a signal region surrounding the beam.

At FRIB, the 6WC was mounted in a beam of ^86^Kr^+26^ ions at 2.75 MeV/u. Currents from a few particles per second (pps) to 5×10^5^ pps were independently determined, with an estimated uncertainty of ~10%, by silicon strip detector for rates ≤ 100 pps, a microchannel plate (MCP) for the 100–1000 pps range, by calibrated beam attenuators for the 1,000 to 25,000 pps interval, and a Faraday cup for rates > 25,000 pps. Beam image data were acquired in 1 s frames, using 6–200 μm thick PM scintillators at rates of ~5000 pps to 520,000 pps and rates down to single particles using the HM scintillator. The total range of a 236 MeV ^86^Kr^+26^ ion in PM, calculated from the SRIM code [23], is 38 μm. Figure 2 shows background-subtracted images and ADC spectra for 6 μm thick PM at 520,000 pps (top) and 51,000 pps beam current (bottom). The ADC spectra were acquired within a limited region surrounding the primary beam. For this test, the 6 μm thick PM scintillator was expected to allow most of the beam to pass through the scintillator (see Section 3.2). The average ADC amplitudes, determined by the restricted Gaussian fits, are 67 ± 0.8 and 7 ± 0.7 respectively. The ratio of the ADCs at 520,000 pps to 51,000 pps is 9.6 ± 0.1 and closely tracks the beam current ratio of 10.2 and, therefore, closely scales to the relative beam currents, where the small differential is from the frame-to-frame signal fluctuations.

**Figure 3:**
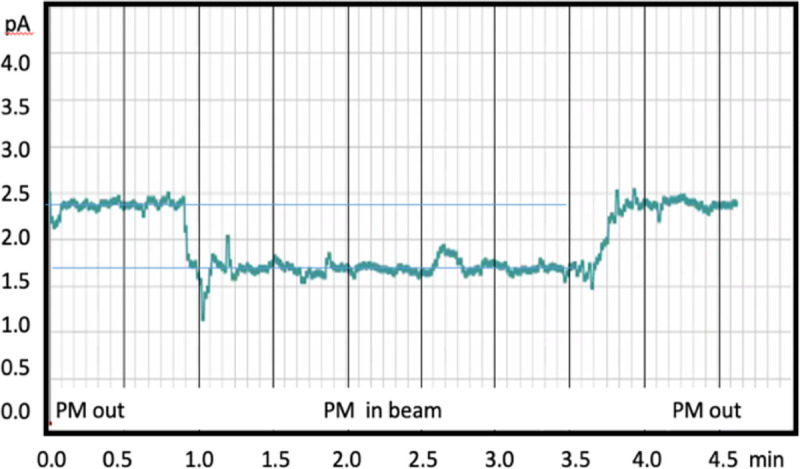
Beam current drop measured by a downstream Faraday Cup when a 6 μm thick PM scintillator is positioned in the beam. Data provided by FRIB ReA3 beam control room.

**Figure 4:**
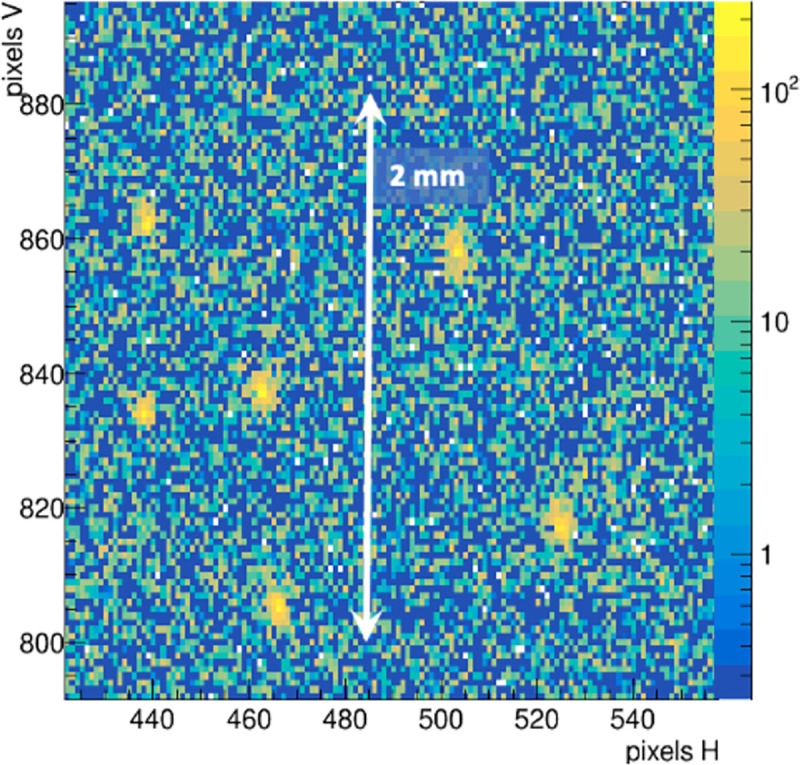
Isolated, single ^86^Kr^+26^ ions detected in HM scintillator at an ultra-low current beam of ~6 pps.

A test of real-time (1 Hz update rate) beam profiling was conducted using a very low current 4,900 pps beam that was translated in a grid pattern by the FRIB control-room operator. The 6WC image data was streamed to online software that analysed and displayed the XY centroids, RMS width and signal strength [22]. Transmission of the heavy ion beam current through a 6 μm thick PM scintillator was measured by a Faraday cup located a few cm downstream of the 6WC. Figure 3 reports this beam current with the scintillator before, during, and after the PM was placed in the beam. With the scintillator in place, the current drops from ~2.4 pA to ~1.7 pA, allowing approximately 71% ion transmission. The HM scintillator was tested in the ion beam for very low currents down to single particles. Figure 4 shows a single frame with a beam current of ~6 pps. Isolated ion hits are readily detected. Integration of the total signal for hundreds of such hits acquired in 60 frames yields a single particle light yield that is then used to normalize the total beam current in each of the data runs. Previous results [22], using this normalization technique, indicate that the HM scintillator has a linear response to beam currents for high-LET ^86^Kr^+26^ ions at 2.75 MeV/u from single particles to at least 500,000 pps, which was the maximum particle beam rate available at FRIB on the day of this experiment.

### 3.2 NDRL Beam Tests

Two beam tests, a year apart, were conducted at NDRL in which first the 6WC was staged, followed by the prototype FSBM a year later. NDRL provided a high intensity 8 MeV electron beam that allowed for measurement of scintillator response and degradation from clinically relevant radiation dose rates, extending to well above the benchmark of a FLASH compatible mean dose rate of 40 Gy/s. The beam, collimated through a 0.5 cm diameter bore in 2.5 cm thick steel, delivered 1–2 ns pulses at 5–30 Hz repetition rates up to 3.3 nC per pulse. The charge was measured with a Faraday cup positioned at the beam exit. Dose per pulse, DPP, was determined from the product of the beam intensity and energy loss: $DPP\;=\phi \frac{dE}{dx}$ where ϕ is charge current density in units of nC/cm-s and $\frac{dE}{dx}$ is the electron stopping power [15] in units of MeV cm^2^/kg. The beam profile was closely approximated by a Gaussian and therefore the diameter was defined as the FWHM of the beam spot. The corresponding charge under the FWHM was taken to be 76% of the total measured. The absolute dose was validated using pre-calibrated Gafchromic films in place of the scintillator targets. In the first test beam the DPP ranged from 0.01–2 Gy and the maximum delivery rate reached 10 Gy/s. In the second test beam the DPP extended from 0.23–7.8 Gy at 30 pps with a maximum dose rate of 234 Gy/s. These tests were consistent with, and in fact went well beyond, a benchmark FLASH electron beam delivering a DPP = 0.4 Gy at a pulse rate of 100 Hz [24]*.*

#### HM scintillator:

**Figure 5:**
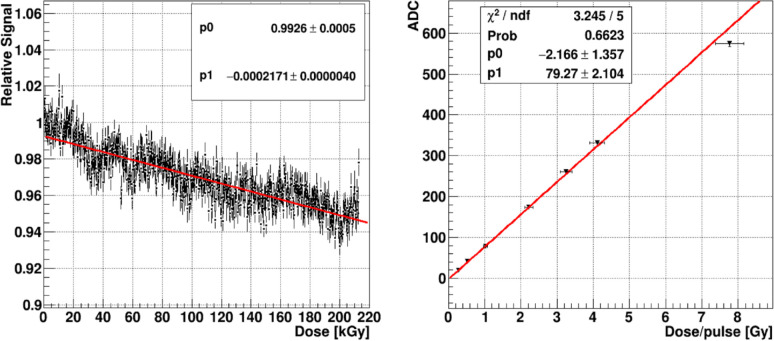
Left panel is normalized HM scintillator relative signal, R (see text) during extended irradiation. The right panel shows the HM signal vs DPP. Red lines are linear fits.

Two measurements demonstrate the HM radiation hardness and response linearity. Figure 5 (left) shows the HM scintillator signal vs the cumulative dose up to 212 kGy, which was delivered continuously in 15 minutes. Such a large dose delivered in such a short time period represents an acceleration factor on the order of 10^5^ and does not allow any time for radiation damage recovery which we have observed as significant in other scintillators such as PM. As a general rule, huge acceleration factors such as described above, often present unrealistically large worst case scenarios, because they can produce multi-particle radiation damage mechanisms that under normal circumstances of reduced irradiation have a much lower probability of occurring.

The Y axis data are $R=\frac{A(t{)}_{beam\;}}{A(0{)}_{beam\;}}/\frac{A(t{)}_{control\;}}{A(0{)}_{control\;}}$ where $A_{beam,}\;A_{control\;}$ are respectively the average ADC signals in the primary beam and in a control region at time t from the start of irradiation. The control region is completely outside of the primary beam and receives less than 0.1% of the beam current. This ratio of ratios, R, removes most of the time dependent beam current drift and periodic fluctuations, although a remnant remains. The signal loss obtained from a linear fit over the 212 kGy cumulative dose is −0.02% (±0.0004%)/kGy.

The signal from a 105 μm thick HM scintillator was measured over a dose range that spanned 0.23–7.8 Gy DPP delivered at an average dose rate of 6.9–234 Gy/s. This range nicely brackets the FLASH dose rates. There is a completely linear response over this relevant region as shown in Figure 5 (right). The dominant source of error derives from ~5% beam current fluctuations. The beam pulse charge could only be measured before or after signal measurement since the Faraday cup interrupted the beam to the FBSM.

#### PM scintillator:

A briefer radiation degradation measurement was also conducted for the PM scintillator in three short runs of 3 kGy each, for a 9 kGy total dose. The PM exhibited no observable loss at a dose rate of 10 Gy/s, in air, for a total dose of 9 kGy. The signal remained stable to within 0.3% RMS. At very high dose rates of ~300 Gy/s and in vacuum, degradation was observed [22]. But we have also shown that in a vacuum, radiation damage recovery does not occur, whereas in air rapid radiation damage recovery was observed.

**Figure 6:**
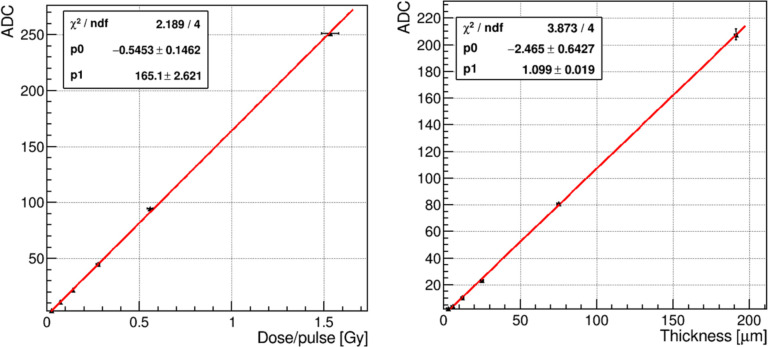
Left panel PM signal vs DPP. Right panel: PM signal vs scintillator thickness. Red lines are linear fits.

The signal from a 200 μm thick PM scintillator was measured over a dose range that spanned DDP ranging from 0.1 Gy to 1.5 Gy. Figure 6 (left) shows the signal ADC counts vs DPP. The PM signal increases linearly. The PM signal is also linear with respect to scintillator thickness over a range from 3–200 μm, shown in Figure 6 (right).

### 3.3 MIBL Beam Test

**Figure 7:**
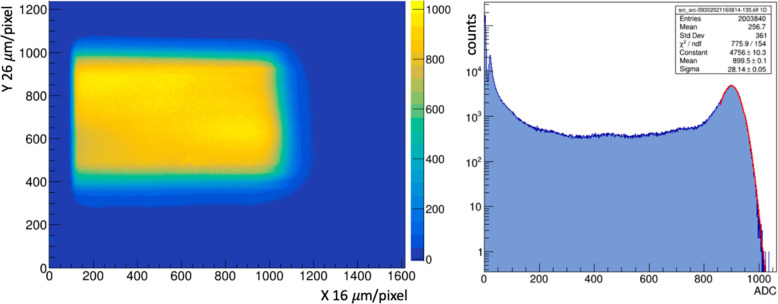
(left) 10 ms (~3 Gy) exposure of a scanning 5.4 MeV proton beam in the 6WC on a 200 μm PM scintillator; (right) ADC distribution of hits in the beam raster. The fit to the peak corresponds to the bright rectangular region in the left panel.

The response of thin PM type scintillators to FLASH intensity proton beams was measured using the 6WC prototype staged at the MIBL. A continuous 5.4 MeV proton beam (roughly 3–5 mm FWHM) of 10 nA was horizontally and vertically swept across an aperture at about 2 kHz and 200 Hz, respectively, to produce a current density at the scintillator target of ~4 nA/cm^2^ and a dose rate ~300 Gy/s. Figure 7 (left) shows the image of the beam on 200 μm thick PM obtained with a 10 ms frame exposure and an average dose of ~3 Gy/frame. The pixel amplitude spectrum for this raster region from Figure 7 (right) is 809 ± 160 ADC counts (~270 photoelectrons(PE)/pixel), where the error reflects 20% fluctuations in the beam current.

### 3.4 Prototype FBSM in a Varian Linac

**Figure 8:**
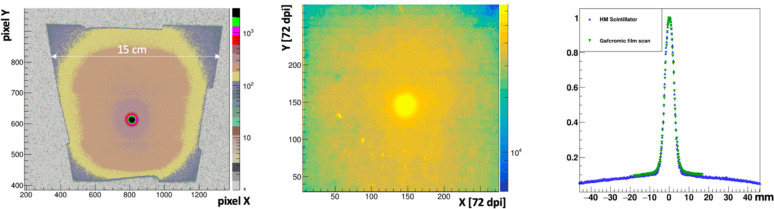
(left) Camera image in the sensor pixel coordinate system of 16 MeV, 0.17 Gy dose electron beam on HM scintillator. (center) A Gafchromic film image of the same beam after a 20 Gy isocenter-equivalent dose exposure. (right) The projection of the beam along the X-axis for an averaged 10 pixel wide Y-axis band for the Gafchromic film and prototype FBSM image data.

The prototype FBSM with HM scintillator was tested at the UMH using a Varian TrueBeam linac. The isocenter-equivalent dose rates corresponded to conventional (i.e., non-FLASH) treatment modes and ranged from 3–10 Gy/min and electron energies ranged from 6–16 MeV. Figure 8 (left panel) shows the background-subtracted image for a 16 MeV beam delivering 0.17 Gy (1 s exposure). A false-color palette reveals the primary electron beam and also the structure of the non-uniform mass profile of the collimator, which generates bremsstrahlung x-rays that penetrate to the scintillator. The trapezoidal shape of the square target is generated by the perspective view of the camera. The center panel shows an image produced by this same beam in a 2 minute exposure to Gafchromic EBT-XD film, corresponding to a 20 Gy dose. The relative average signal amplitude for a 10 Y-pixel wide band projected along the X coordinate and passing through the beam center is shown in Figure 8, right panel, for both the Gafchromic film overlayed with the prototype FBSM image. The Gafchromic data were corrected for the film’s non-linear response [16]. The residual difference in the two distributions has an RMS spread of 0.5%, indicating that the spatial resolution of the prototype FBSM is compatible with the < 25 μm manufacturer’s specification of the Gafchromic film [25].

## 4 DISCUSSION

### 4.1 Overall

Initial beam tests of the two FBSM prototypes established important attributes necessary for FLASH RT applications. The combined beam tests at four facilities allowed performance testing over a broad dynamic range of signals generated from single particles to FLASH-level dose rates. The FRIB beam test demonstrated that real-time image processing and beam analysis could be achieved using a machine-vision camera. All the required operations: image background subtraction, perspective transforms, beam profiling and dosimetry were performed in a continuous data flow model with no dead time using the DAQ computer CPU and on-board memory. A similar set of processing algorithms coupled to a data stream generated by a 10–200 times faster camera is being implemented in FPGA firmware to achieve image processing at a 100 μs output rate. Most of this time is consumed by the time required to transfer data from the sensor to the FPGA. We have demonstrated with a different camera selected for proton FLASH-RT, operating at an ultrafast frame rate of 20,000 fps (50 μs/frame), that we can perform a full analysis of beam position, beam shape, and beam dose within < 1 μs.

### 4.2 Dosimetric Response

#### HM Scintillator:

In the ^86^Kr^+26^ beam at FRIB, the HM scintillator was staged in the 6WC prototype. A linear response extending from single particles to 5×10^5^ pps, spanning five orders of magnitude was measured [22]. Results obtained from UMH in Section 3.4, however, extend the linearity of response to clinical dose levels. Figure 8 (right panel) shows that signal amplitude tracks the linearized reference Gafchromic film measurement over nearly two orders of magnitude, from about 5% in the tails to 100% relative amplitude at the peak where the corresponding isocenter equivalent dose is 0.17 Gy.

#### PM Scintillator:

The 6 μm thin PM scintillator was shown to be transmissive to even very heavily ionizing beams. This ultra-thin scintillator material might also be used for proton FLASH applications. Figure 7 shows, for example, that a 3 Gy/frame dose yields 270 PE/pixel. In a benchmark FLASH beam, an 8 Gy dose is delivered by a scanning 5 mm diameter proton pencil beam with a dwell time of 1 ms per beam spot location *[4]*. A FLASH beam monitor running at 20 kHz (50 μs per frame) would capture 20 time slices of this dose with a corresponding signal of (1/20 × 8Gy/3Gy × 270[PE]) = 36 PE/pixel. In the 6WC device used for Figure 7, the total photoelectron signal is obtained by integration of the beam spot over several thousand pixels. Conversion of this 6WC signal into the equivalent for a prototype FBSM device is contingent upon the specific optics, pixel dimensions and quantum efficiency of the sensor. The dosimetric uncertainty from photo-statistics, estimated from sqrt(PE)/PE is expected to be under 1% per time slice, more than adequate to generate an immediate out-of-tolerance beam interrupt for FLASH applications. The uncertainty from systematic contributions, e.g., spatial non-uniformities of light collection, beam width determination, integration of residual readout noise, etc. is the focus of ongoing investigation.

### 4.3 Radiation Tolerance and Calibration

Section 3 reports that the HM signal remains resistant to high dose rates with a net relative decrease of only 0.02%/kGy over a total dose of 212 kGy delivered in 15 minutes. The PM scintillator was stable to better than ±0.4% RMS for a total dose of 9 kGy. These experiments delivered a highly accelerated dose with respect to any clinical application. These materials, and especially the HM scintillator, could be expected to be irradiated at daily clinical doses for a year or more with less than 1% total signal loss, without even allowing for any partial recovery from room temperature thermal annealing that occurs in inorganic scintillators over a time scale of days [26]. Additionally, an internal, self-monitored UV LED-based calibrator mechanism is used to monitor the long-term stability. Periodic illumination of the scintillator produces an image that can be measured against a reference. Differential light yields between the reference and the current image are then used to generate a spatially dependent signal correction matrix. This matrix records the spatially dependent signal amplitude corrections registered by the calibration procedure. A significantly measurable average global signal loss would be used to indicate scintillator replacement. Ongoing efforts are underway to establish the precision of these corrections, but the precision is expected to be at least as good as the 0.4% uncertainty set by the HM scintillator measurement in Figure 5 (left). Nonetheless, degradations of more than 1% would likely trigger scintillator replacement. Two factors suggest, however, that a maximum dose at which scintillator replacement is required might be very high: First, the scintillator is positioned at the beam exit where the dose rate is less than at the isocenter [27]. Second, previous measurements on the PM scintillator by our group have shown that the radiation damage tends to partially recover in air over a time scale of hours [22]. How this dose tolerance translates to material longevity in a clinical or research application will ultimately depend on the specific and currently unknown parameters of clinical usage, but it is certainly possible that these scintillators could last a year or more before suffering a 1% loss in photon emission efficiency and requiring replacement.

### 4.4 Spatial Resolution

**Figure 9:**
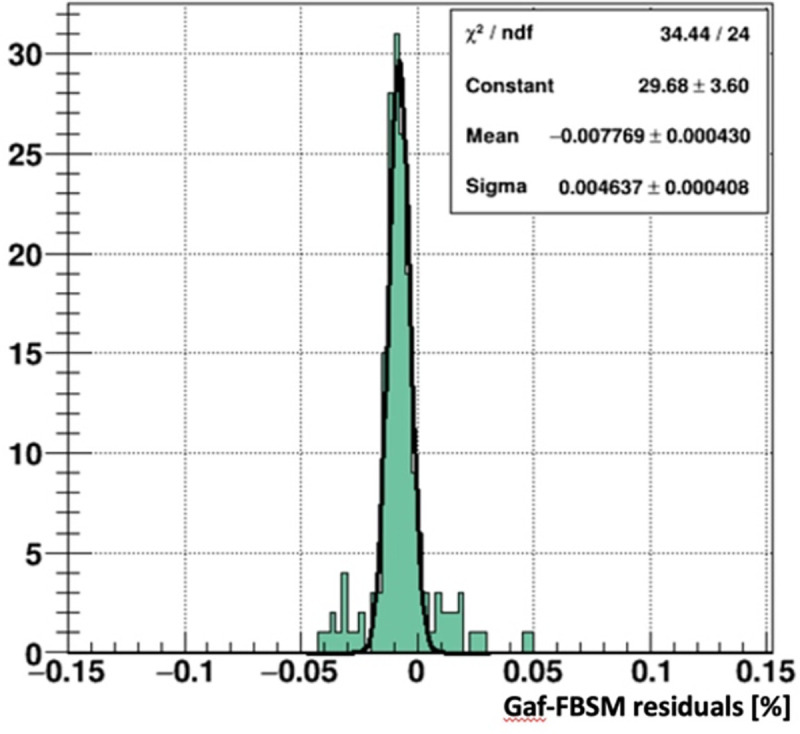
Residuals of the Gafchromic film and prototype FBSM profile distributions in Figure 8, right panel.

The spatial resolution of the prototype FBSM using an HM scintillator was measured against Gafchromic EBT-XD films. The beam profile plots in Figure 8 (right panel) were compared in two ways: In the first, they were fit with double Gaussian distributions of the form: $f(x)=A_{n}e^{{\left(\frac{x}{2\sigma_{n}}\right)}^{2}}+A_{w}e^{{\left(\frac{x}{2\sigma_{w}}\right)}^{2}}$ where the subscripts n and w denote the narrow primary beam and the wide tails, respectively. The average of the fit results for HM and Gafchromic distributions in Figure 8 are $s_{w}=30.0\pm 0.5\;mm$ and $s_{n}=2.14\pm 0.03\;mm$. That is, the two distributions can be described by the same functional form where the fit parameters agree to within 1.4%. However, a direct comparison of the two projective plots was obtained from the histogram of their point by point differences. This residual distribution is shown in Figure 9. The RMS spread of 0.5%, indicates that the spatial resolution of the prototype FBSM is compatible with the < 25 μm manufacturer’s specification [25] for Gafchromic film.

### 4.5 Application in FLASH Beam Monitoring

One of the important functions of the FBSM is to assert a beam interlock when the irradiation deviates from the dose delivery program. A relevant regulation, IEC 60601–2-1 [28], indicates that no more than 10% of the prescribed dose is delivered after interlock assertion. This demands both dosimetric precision and sufficient data processing of the FBSM. The FBSM signal response using the HM scintillator was shown to be linear and stable to a few percent or better over many orders of magnitude, specifically for clinical dose exposure (Section 4.2). A beta version of the FBSM data acquisition and analysis has been implemented in firmware for real-time readout of the camera. The firmware performs all necessary operations including background subtraction, beam finding and calculation of the centroids, beam width and integrated signal. The total response time of the readout algorithm was measured with the Vivado FPGA logic analyser (Xilinx, Advanced Micro Devices, Inc.) to be approximately 50 μs, although there is net 100 μs lag of the analysis output from the start of the data frame due to a 50 μs delay in the initial frame’s data processing. The total latency budget is consumed by data transfer, image processing and analysis. The bulk of this time is consumed by data transfer. The image processing and analysis stages are conducted in under 1 μs. The IEC 60601–2-1 regulation applies to pulsed electron beam delivery with as few as 10 pulses per treatment. Proton beams from isochronous cyclotrons deliver quasi-continuously at a dose rate, R. The residual dose, therefore, would not exceed 0.1 R*dT_i_, where dT_i_ is the 100 μs required by the FBSM to assert a beam interlock. Assuming a standard FLASH dose rate of R=100 Gy/s, the residual dose is 0.01 Gy. This means that a dose as small as 0.1 Gy could be prescribed at a 100 Gy/s dose rate, and therefore the FBSM would be in regulatory compliance with any FLASH application.

## 5 CONCLUSIONS

We have presented the design and test results of a scintillator based beam monitor for FLASH radiation therapy. The FBSM described here is designed to provide real-time precise spatially dependent dosimetry of beam profiles without significantly degrading the beam quality, and over a large area. Prototype devices have been staged in particle beams at currents of single particles up to high FLASH-level dose rates, using both continuous ion beams and pulsed electron beams. Transverse beam profiling has been demonstrated for currents extending from single particles to 10 nA currents using two novel scintillators. A linear dose response has been demonstrated. In a clinical electron beam test, the spatial resolution was comparable to radiochromic films. However, radiochromic films cannot provide real-time analysis or dosimetry. The two novel scintillators are radiation tolerant under nominal and accelerated clinical exposure, and real-time analysis has been demonstrated. Data processing and analysis are currently being implemented in firmware for 10–20 kHz frame rates for continuous proton beams with better than 1 mm spatial resolution and for pulsed electron beams for pulse rates of 400 Hz at < 100 μm spatial resolution. Operation for electron beam pulse rates extending to 1000 Hz is also anticipated but at reduced spatial resolution.
